# Comparative effectiveness of neoadjuvant chemotherapy plus surgery versus concurrent chemoradiotherapy in stages IB2 to IIB of cervical cancer: a meta-analysis

**DOI:** 10.3389/fonc.2024.1426002

**Published:** 2024-06-24

**Authors:** Yue Gao, Huali Wang, Meng Jiang

**Affiliations:** Department of Gynecology, Dalian Women and Children’s Medical Center (Group), Dalian, Liaoning, China

**Keywords:** cervical cancer, neoadjuvant chemotherapy, chemoradiotherapy, surgery, meta-analysis

## Abstract

**Objectives:**

To assess the comparative efficacy of neoadjuvant chemotherapy followed by surgery (NACT+S) versus concurrent chemoradiotherapy (CCRT) for patients with cervical cancer stages IB2 to IIB.

**Method:**

An exhaustive literature search was conducted up to November 2023 in databases including PubMed, Embase, Web of Science, and the Cochrane Library, focusing on disease-free survival (DFS) and overall survival (OS). Data were analyzed using STATA version 15.

**Results:**

The meta-analysis included data from two randomized controlled trials and eight retrospective cohort studies, totaling 2,879 patients with stages IB2 to IIB cervical cancer. Pooled data showed no significant difference in OS [hazard ratio (HR) 0.71, 95% confidence interval (CI): 0.51 to 1.00, p = 0.052] and DFS (HR 0.65, 95% CI: 0.38 to 1.14, p = 0.132) between NACT+S and CCRT. Subgroup analysis revealed that NACT+S provided a better OS in Asian populations, retrospective cohort studies, TP regimen chemotherapy, and multivariate analysis.

**Conclusion:**

The findings indicate that CCRT and NACT+S are comparably effective for treating cervical cancer stages IB2 to IIB. Notably, in specific subgroups such as Asian patients and those receiving the TP regimen, NACT+S appears to enhance OS.

## 1 Introduction

Cervical cancer ranks as the fourth most common and fatal cancer among women globally (1), with approximately 604,127 new cases and 341,831 deaths in 2020. The age-standardized incidence and mortality rates were reported at 13.3 and 7.2 per 100,000 women-years, respectively (95% CI for both ranges slightly) (2). In response, the World Health Organization launched the Cervical Cancer Elimination Initiative in 2020, aimed at accelerating the eradication of this disease. Prominent risk factors include smoking, oral contraceptive use, early sexual activity, multiple sexual partners, sexually transmitted diseases, certain autoimmune diseases, and chronic immunosuppression (3, 4).

The 2018 update to the International Federation of Gynecology and Obstetrics (FIGO) system refined cervical cancer staging to include subdivisions of Stage IB (IB1, IB2, IB3), with Stage IB3 (FIGO 2018) now corresponding to the earlier Stage IB2 (FIGO 2009), ensuring clinical staging consistency. For women with (FIGO 2018) Stage IB2 or higher, the standard treatment is chemoradiotherapy, combining cisplatin-based chemotherapy with concurrent radiotherapy over seven weeks to enhance the cancer cells’ sensitivity to radiation, thus improving outcomes. However, surgery may be considered under specific conditions in locally advanced cervical cancer (LACC) (Stages IB2 to III): if there is no complete remission within two to three months post-chemoradiotherapy and the tumor remains operable; if prior treatments reduce the tumor to a surgically removable size; or following neoadjuvant chemotherapy (NACT), especially in regions with limited radiotherapy access. The efficacy of NACT over surgery alone or combined with chemoradiotherapy continues to be evaluated (5, 6). Despite a five-year overall survival (OR) rate of around 70% for these stages, patients’ quality of life is often affected (7), with radiotherapy notably impacting sexual health in younger, premenopausal women (8).

In locally advanced or Stage IIB cervical cancer, neoadjuvant chemotherapy prior to surgery (NACT-S) has shown potential in reducing tumor size and addressing subclinical lesions (9). However, consensus on the efficacy of this treatment is not fully established, with studies yielding mixed results. Chang et al. reported a 2-year survival rate of 81% (95% CI: 71%-91%) and an estimated 5-year survival rate of 70% for patients undergoing NACT-S (10). Uegaki et al. suggested that NACT-S followed by radical hysterectomy might cure approximately 70% of patients with Stages IB2 to IIB cervical cancer (11). Despite these findings, the definitive benefits of NACT-S are still under investigation. Comparative studies, such as those by Yoshida et al., have noted significantly longer progression-free and OS in patients receiving surgery post-neoadjuvant chemoradiation compared to those receiving chemoradiation alone, with p-values of 0.027 and 0.017 respectively (12).

## 2 Objectives

The ongoing debate underscores the need for further research to clarify the comparative prognosis of NACT-S and concurrent chemoradiotherapy (CCRT). This study aims to assess the comparative efficacy of NACT followed by surgical intervention (NACT+S) versus CCRT in patients with cervical carcinoma at stages IB2 to IIB.

## 3 Methods

### 3.1 Search strategy

The study protocol was registered with the International Prospective Register of Systematic Reviews (PROSPERO, CRD 42023482991). A comprehensive literature search up to November 2023 was conducted across databases such as PubMed, Embase, Web of Science, and the Cochrane Library to identify relevant studies. Additional sources including references in found articles and related reviews were examined to ensure the capture of all pertinent publications. Search terms included “cervical cancer” or “uterine cervical neoplasms”, and “chemoradiotherapy” or “chemoradiation”, without language restrictions. The detailed search strategy is available in **Appendix 1**.

### 3.2 Eligibility criteria

Inclusion criteria were: (1) patients with FIGO stage IB2-IIB cervical cancer (the year of FIGO staging used in each article is marked in **Table 1**); (2) study groups undergoing CCRT; (3) control groups receiving NACT+RS; (4) outcomes measured included OS and/or DFS; (5) study designs were either cohort studies or RCTs. Exclusion criteria included: (1) animal studies; (2) studies without available full texts; (3) non-empirical articles such as reviews, meta-analyses, conference abstracts, case reports, and guidelines.

**Table 1:** The main characteristics of the ten included studies.

Study	Year	Country	Design	Control group (NACT-S)		Case group(CCRT)		FIGO stage (year)	NACT	Follow-up (month)	Primary endpoints	Score	Adjuvant therapy
				n	age	n	age						
Yin (13)	2011	China	Retrospective cohort study	187	43	94	47	IB2-IIB (1994)	TP/PVB	82.8	OS,DFS	8	NACT-S group :63(33.7%) patients received postoperative radiotherapy or CCRT
Yang (14)	2015	China	Retrospective cohort study	103	38	141	38	IIB (2009)	TP/TC	67	OS	8	NACT-S group :65(63.1%) patients received patients adjuvant radiotherapy
Gupta (15)	2018	India	RCT	316	50	317	48	IB2- IIB (1994)	TC	58.5	OS,DFS	5	NACT-S group :42(13.3%) patients received adjuvant radiotherapy ; 31 (9.8%)patients received CTRT
Hsieh (16)	2019	China	Retrospective cohort study	39	44	27	54	IB2/IIA/IIB (2009)	PVB	66.2	OS,DFS	7	NACT-S group :16 (41%) patients received adjuvant treatment
Akhavan (17)	2021	Iran	Retrospective cohort study	46	46.41	51	53.06	IB3 /IIA2 (2018)	TP	42	OS,DFS	8	none
Zeng (18)	2022	China	Retrospective cohort study	153	46.6	201	47.11	IB3 /IIA2 (2018)	TC	75	OS,DFS	9	NACT-S group :15 (9.8%) patients received postoperative radiotherapy; 10 (6.5%) patients received postoperative chemoradiotherapy.
Zhang (19)	2022	China	Retrospective cohort study	53	46	49	46	IB2/IIA2 (2009)	TC	57	OS	9	NACT-S group :16 (30.2%) received patients Radiotherapy, 25 (47.2%) patients received Chemoradiotherapy
Kenter (20)	2023	Europe	RCT	314	46	312	47	IB2-IIB (2009)	TC	104.4	OS	6	NACT-S group :Adjuvant (chemo)radiotherapy was recommended in the case of proven lymph node metastases, parametrial infiltration or positive surgical margins.
Li (21)	2023	China	Retrospective cohort study	175	49.14	175	49.1	IB2/IIA2 (2009)	TP	36	OS,DFS	9	NACT-S group :Postoperative adjuvant radiation therapy with or without concurrent chemotherapy was applied

### 3.3 Data collection and quality assessment

Initial screenings of titles and abstracts were followed by full-text reviews for articles meeting the inclusion criteria. Two researchers independently extracted data using predefined forms, focusing on OS, DFS, and essential study characteristics including authorship, publication year, geographical location, study design, FIGO stage, NACT methodology, and follow-up period. Data on OS and DFS not directly available were extracted from Kaplan-Meier survival curves using Engauge Digitizer 4.1. The methodological quality of studies was assessed using a modified 7-point Jadad Score for randomization, allocation concealment, double-blinding, and withdrawals/dropouts, with scores 1–3 indicating high risk of bias and 4–7 indicating low risk. The Newcastle-Ottawa Scale (NOS) further evaluated the selection, comparability, and outcome of studies, categorizing quality as low (0–3), moderate (4–6), or high (7–9).

### 3.4 Statistical analysis

Statistical analyses were performed using Stata (version 15.0) and Review Manager (version 5.3.3) for literature quality evaluation. Pooled data provided summary hazard ratios (HRs) and 95% CIs for OS and DFS. Risk ratios (RRs) and corresponding 95% CIs compared adverse events between the NACT+S and CCRT groups. Cochran’s Q test and the *I^2^* statistic assessed study heterogeneity. An *I^2^* value above 50% prompted the use of a random-effects model, while values below 50% utilized a fixed-effects model. Subgroup analyses were based on study design, region, average follow-up period, NACT regimens, and data sources. Egger’s test evaluated publication bias, and sensitivity analyses applied the “remove one study” method to determine the impact of individual studies on overall outcomes. A p-value less than 0.05 was considered statistically significant.

## 4 Results

### 4.1 Study selection and quality assessment

**Figure 1:**
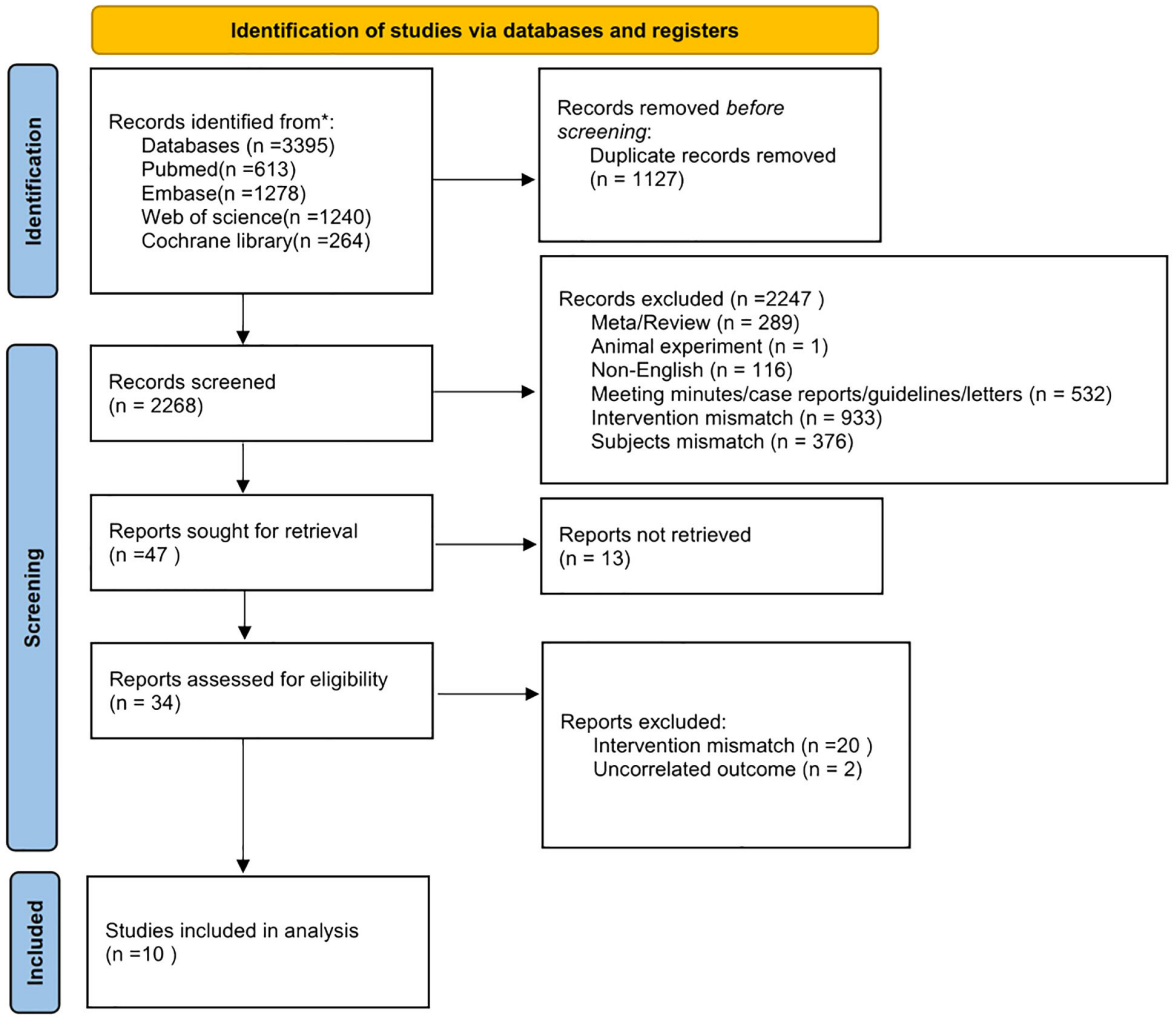
Flow diagram of the entire research literature.

The literature search and screening, summarized in **Figure 1**, identified 3,395 articles from databases including PubMed, Embase, Web of Science, and the Cochrane Library. After removing 1,127 duplicates, 2,268 articles were screened, leading to the exclusion of 2,247 articles for various reasons: meta-analyses/reviews (289), animal studies (1), non-English articles (116), meeting minutes/case reports/guidelines/letters (532), intervention mismatch (953), subject mismatch (376), and unrelated outcomes (2). Ultimately, ten articles met the inclusion criteria for this study.

### 4.2 Study characteristics and quality assessment

The ten selected studies (12–21), comprising eight retrospective cohort studies and two RCTs, involved a total of 2,879 patients aged 38 to 56.9 years, diagnosed with cervical cancer stages IB2 to IIB. These studies originated from Asia and Europe and were assessed for quality using the Cochrane Collaboration’s risk of bias tool. Studies scoring ≥7 for cohort studies and ≥5 for RCTs were classified as high quality, as detailed in **Table 1**.

### 4.3 Pooled analysis for OS

**Figure 2:**
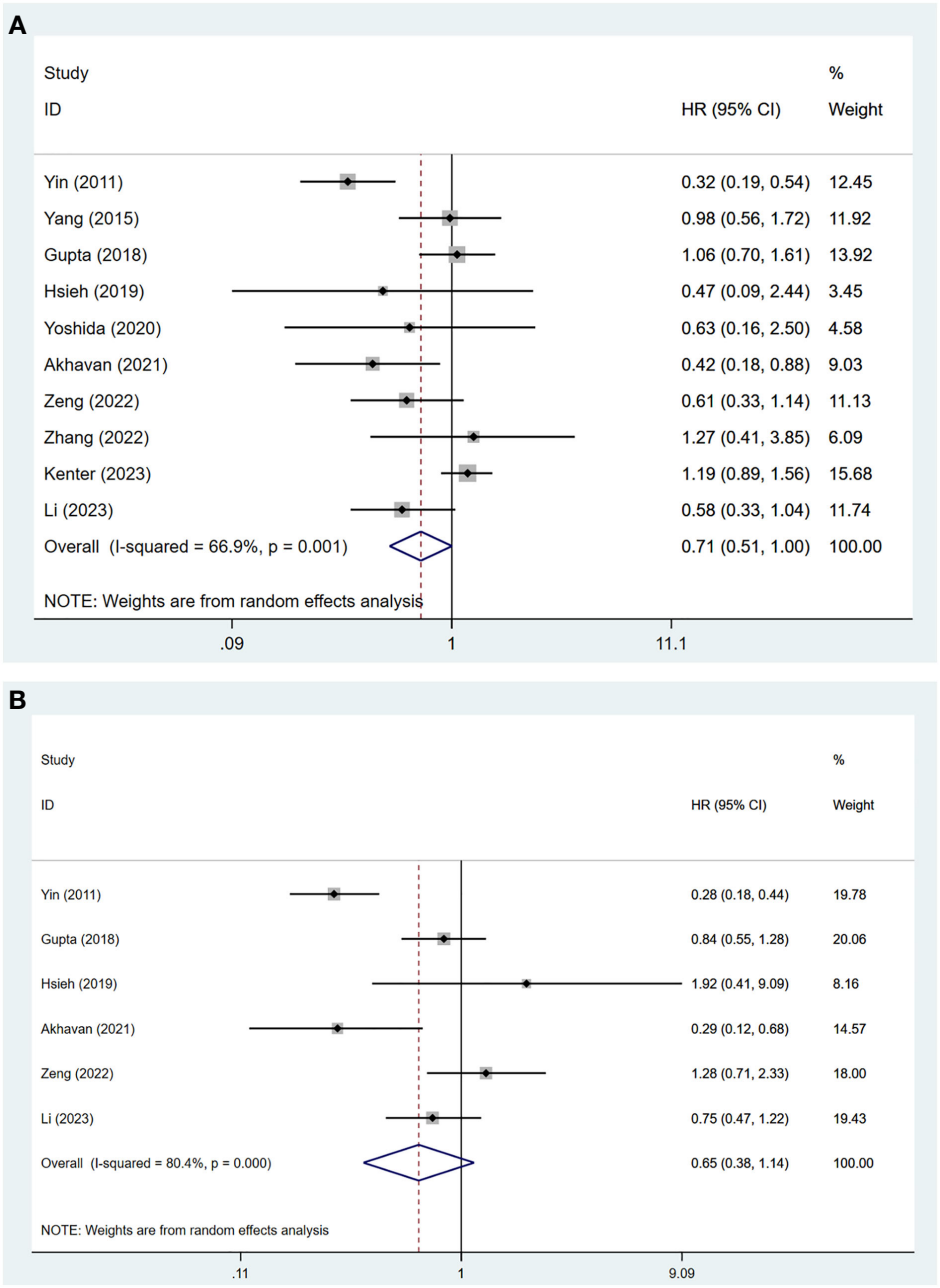
Forest plots for the **(A)** OS and **(B)** DFS.

The meta-analysis of these ten studies showed substantial heterogeneity (*I^2^* = 66.90%, p = 0.001), necessitating a random-effects model. The results indicated no significant difference in OS between patients treated with NACT+S and those undergoing CCRT (HR = 0.71, 95% CI: 0.51 to 1.00, p = 0.052) (**Figure 2A**).

### 4.4 Subgroup analysis for OS

Subgroup analyses explored sources of heterogeneity and yielded several findings: In the Asian population, OS was significantly higher with NACT+S compared to CCRT (HR = 0.65, 95% CI: 0.46 to 0.92, p = 0.014). Retrospective cohort studies also showed improved OS with NACT+S (HR = 0.58, 95% CI: 0.42 to 0.81, p = 0.001), a result not seen in RCTs. NACT using the TP regimen was associated with better OS outcomes (HR = 0.52, 95% CI: 0.33 to 0.83, p = 0.006). Follow-ups longer than 60 months did not show significant variation in OS. Multivariate analysis further confirmed superior OS with NACT+S (HR = 0.46, 95% CI: 0.33 to 0.63, p < 0.001), whereas univariate analysis and data extracted via software showed no significant differences. These detailed findings are elaborated in **Table 2**; **Supplementary Figures** in the **Appendix**.

**Table 2:** The results of pooled HR for OS and DFS.

	No.	HR (95%CI)	*p*	Heterogeneity *I* ^2^(%) P
OS				
overall	10	0.71 (0.51-1.00)	0.052	66.90%, 0.001
Subgroup				
Region				
Asia	9	0.65 (0.46-0.92)	0.014	53.50%, 0.028
Europe	1	1.19 (0.90-1.58)	0.224	–
Design				
Retrospective cohort study	8	0.58 (0.42-0.81)	0.001	36.00%, 0.141
RCT	2	1.15 (0.91-1.45)	0.246	0, 0.652
NACT regimens				
TC	4	1.04 (0.80-1.35)	0.776	21.10%, 0.284
PVB	1	0.47 (0.09-2.45)	0.370	–
FP	1	0.63 (0.16-2.49)	0.510	–
TP	2	0.52 (0.33-0.83)	0.006	0, 0.518
Mean follow-up period (month)				
≥60	6	0.68 (0.40-1.15)	0.149	76.20%, 0.001
<60	4	0.75 (0.47-1.21)	0.234	51.20%, 0.105
Data sources				
Multivariate Analysis	4	0.46 (0.33-0.63)	<0.001	9.90%, 0.344
Univariate Analysis	2	1.15 (0.91-1.45)	0.246	0, 0.652
Engauge Digitizer	4	0.92 (0.59-1.45)	0.725	0, 0.730
DFS				
overall		0.65 (0.38-1.14)	0.132	80.40%, <0.001
Subgroup				
Design				
Retrospective cohort study	5	0.62 (0.31-1.25)	0.184	82.40%, <0.001
RCT	1	0.84 (0.65-1.28)	0.418	–
NACT regimens				
TC	2	0.98 (0.66-1.46)	0.931	22.00%, 0.257
PVB	1	1.92 (0.41-9.04)	0.409	–
TP	2	0.5 (0.20-1.26)	0.141	71.80%, 0.060
Mean follow-up period (month)				
≥60	3	0.79		89.40%, <0.001
<60	3	0.64	0.052	57.90%, 0.093
Data sources				
Multivariate Analysis	4	0.46		9.90%, 0.344
Univariate Analysis	1	1.15	0.014	0, 0.652
Engauge Digitizer	1	0.92	0.224	0, 0.730

### 4.5 Pooled analysis for DFS

A pooled analysis was conducted on data from six studies involving 1,781 patients with cervical cancer stages IB2 to IIB. Due to significant heterogeneity (*I^2^* = 80.40%, p < 0.001), a random-effects model was applied. The results showed no significant difference in DFS between patients undergoing NACT+S and those receiving CCRT (HR = 0.65, 95% CI: 0.38 to 1.14, p = 0.132), as shown in **Figure 2B**.

### 4.6 Subgroup analysis for DFS

Subgroup analyses, considering variables such as study design, NACT regimens, follow-up periods, and data sources, revealed no statistically significant differences in DFS between the NACT+S and CCRT groups. These findings are detailed in **Table 2**.

### 4.7 Sensitivity analysis

**Figure 3:**
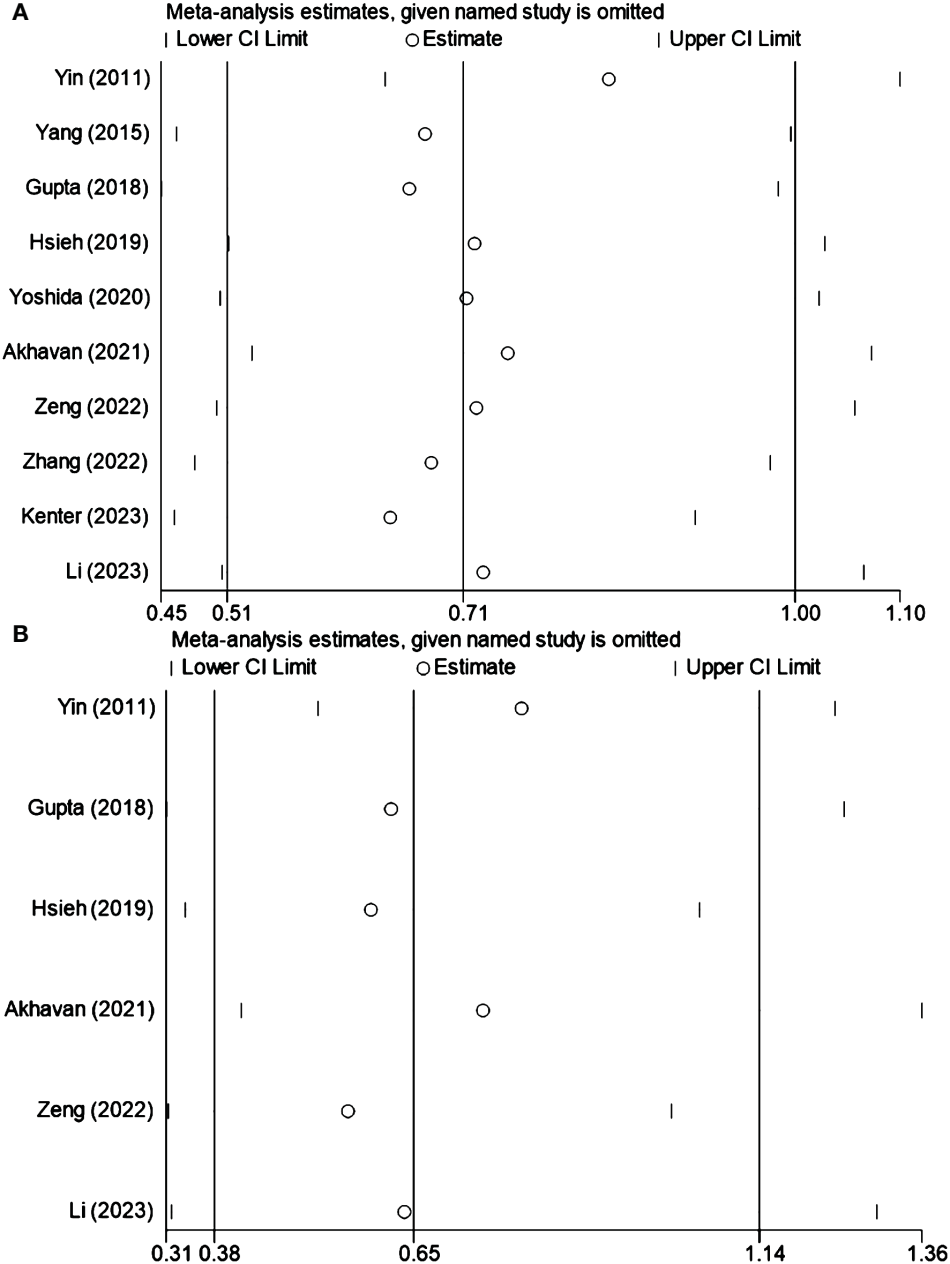
Sensitivity Analysis for **(A)** OS and **(B)** DFS.

Sensitivity analysis was conducted to assess the influence of individual studies on the overall outcomes. This analysis affirmed that no single study markedly affected the composite results, confirming the robustness of the findings. The results are presented in **Figures 3A, B** for OS and DFS, respectively.

### 4.8 Publication bias

With ten studies included in the OS analysis, potential publication bias was evaluated using the Egger test. The results indicated no significant publication bias (p = 0.154), reinforcing the reliability of the OS findings.

## 5 Discussion

### 5.1 Summary of main results

This meta-analysis, incorporating ten studies, compared the effectiveness of NACT+S versus CCRT in patients with cervical cancer stages IB2 to IIB. The overall findings revealed no significant differences in OS and DFS between the two treatments. However, subgroup analyses indicated that NACT+S might improve OS in Asian populations, in retrospective cohort studies, with the TP chemotherapy regimen, and in multivariate analyses. Conversely, other subgroup analyses showed no disparities in OS or DFS across treatment groups.

### 5.2 Results in the context of published literature

The results contrast with those of Cheng et al., who suggested that CCRT might be more effective for cervical cancer at FIGO (2009) stage IB2/IIA2 (22). This discrepancy may be due to this analysis including only two studies with the CCRT regimen and a recent large-scale RCT published in 2023, which may enhance the reliability of our findings. This is consistent with Kenter et al., whose RCT reported no significant difference in 5-year OS between the NACT+S (72%; 95% CI: 66%-77%) and CCRT groups (76%; 95% CI: 70%-80%) (20).

Subgroup analyses from nine Asian studies showed higher OS for the NACT+S group compared to CCRT, a finding not replicated in a single European study. This might reflect limited European data or inherent differences in treatment response due to racial or regional factors. The techniques and strategies of administering CCRT and NACT might also differ across regions. Retrospective cohort studies indicated poorer OS outcomes for the CCRT group, contrasting with findings from RCTs, which benefit from randomized grouping to enhance comparability and minimize bias.

Patients treated with the TP regimen in the NACT+S group exhibited higher OS compared to those undergoing CCRT, suggesting a potential preference for the TP regimen in NACT+S planning. Caution is warranted in interpreting these results, however, due to the limited number of studies focused on specific regimens and the use of multiple regimens within some studies. Finally, while substantial differences were observed in multivariate analysis, univariate analysis and data extracted using Engauge Digitizer did not show similar disparities, possibly due to the limited scope of studies included in these analyses.

This research suggests that NACT+S and CCRT may have comparable therapeutic outcomes. NACT+S can reduce tumor volume, facilitate surgical resection, and potentially decrease subclinical metastasis. It also enables assessment of tumor responsiveness to chemotherapy and prognosis prediction. Considering the risk of vaginal dysfunction associated with CCRT, NACT+S might be preferable for younger patients concerned about sexual health. However, it is critical to acknowledge that NACT+S could obscure high-risk factors, potentially leading to increased recurrence, alongside added side effects and treatment costs. For patients exhibiting high-risk factors post-NACT+S, adjuvant radiochemotherapy remains necessary. Despite these considerations, our analysis of ten studies, nine of which included cases of adjuvant treatment post-NACT+S (**Table 1**), found no differences in DFS and OS between the groups. Given the increased side effects and economic burden associated with overlapping treatments, CCRT may sometimes be more suitable. The role of NACT+S in managing LACC remains controversial but is supported by Li et al.’s findings, which show promising antitumor activity and a tolerable adverse event profile from combining neoadjuvant chemo-immunotherapy with radical surgery, suggesting a novel approach for LACC treatment (23). Continued research is essential to determine the optimal treatment strategy for LACC.

### 5.3 Strengths and weaknesses

This study offers methodological strengths as the first to compare the effectiveness and safety of NACT+S versus CCRT in this patient group, providing clinically significant insights. The inclusion of quality assessments, comprehensive evaluations for publication bias, and meticulous sensitivity analyses enhance the reliability of our findings. Limitations include a predominance of retrospective studies and a regional focus on Asia, which may restrict the generalizability of results. The limited number of RCTs and the regional skew also necessitate cautious interpretation of our findings.

### 5.4 Implications for practice and future research

In summary, while findings suggest comparable therapeutic efficacies of NACT+S and CCRT for cervical cancer stages IB2 to IIB, the predominance of retrospective cohort studies highlights the need for more prospective, high-quality RCTs to confirm these outcomes.

## Author contributions

YG: Data curation, Formal analysis, Methodology, Software, Validation, Writing – original draft, Writing – review & editing. HW: Conceptualization, Methodology, Resources, Writing – review & editing. MJ: Investigation, Validation, Writing – review & editing.
