# Supplementary material for: Comparative effectiveness of neoadjuvant chemotherapy plus surgery versus concurrent chemoradiotherapy in stages IB2 to IIB of cervical cancer: a meta-analysis
Source: Front Oncol. 2024 Jun 24;14:1426002. doi: 10.3389/fonc.2024.1426002 (PMC11228234; doi:10.3389/fonc.2024.1426002)
Supplement: Supplementary file 1 [file DataSheet_1.docx]

**Appendix 1:** Search strategy.

**Cochrane search strategy**

Search Name:

Date Run: 05/10/2023 13:18:23

Comment:

ID Search Hits

#1 MeSH descriptor: [Radiotherapy] explode all trees 10371

#2 (Radiotherap* or Radiation Therap* or Radiation Treatment* or Targeted Radiotherap* or Targeted Radiation Therap* or radiation therapy or X-ray therapy or radioisotope therapy):ti,ab,kw 60164

#3 #1 or #2 60793

#4 MeSH descriptor: [Drug Therapy] explode all trees 180838

#5 (Drug Therap* or Chemotherap* or Pharmacotherap* or pharmacologic therapy):ti,ab,kw 587056

#6 #4 or #5 622754

#7 #3 and #6 35966

#8 MeSH descriptor: [Chemoradiotherapy] explode all trees 2239

#9 Chemoradiotherap* or Radiochemotherap* or Concurrent Chemoradiotherap* or Synchronous Chemoradiotherap* or Concurrent Radiochemotherap* or Concomitant Chemoradiotherap* or Concomitant Radiochemotherap* or Chemo-Radiotherapy or Chemoradiation 10564

#10 #8 or #9 10564

#11 #7 or #10 39390

#12 MeSH descriptor: [Uterine Cervical Neoplasms] explode all trees 2980

#13 Uterine Cervical Neoplas* or Cervical Neoplas* or Cervix Neoplas* or Cervical Cancer* or Uterine Cervical Cancer* or Cancer of Cervix or Cervix Cancer or uterine cervix tumor or cervical tumo* or cervix neoplasm* or cervix tumorigenesis or cervix uteri tumor or neoplastic cervical or neoplastic cervix or uterine cervical neoplasm* or uterine cervical tumor or uterine cervix neoplas* or uterine cervix tumo* 8848

#14 #12 or #13 8848

#15 MeSH descriptor: [Neoadjuvant Therapy] explode all trees 2205

#16 Neoadjuvant Therap* or Neoadjuvant Treatment* or Neoadjuvant Chemoradiotherap* or Neoadjuvant Chemoradiation Therap* or Neoadjuvant Chemoradiation Treatment* or Neoadjuvant Chemoradiation* or Neoadjuvant Chemotherap* or Neoadjuvant Chemotherapy Treatment* or Neoadjuvant Systemic Therap* or Neoadjuvant Systemic Treatment* 11892

#17 #15 or #16 11892

#18 #11 and #14 and #17 264

**EMbase search strategy**

| No. | Query | Results |
| --- | --- | --- |
| #18 | #11 AND #14 AND #17 | 1278 |
| #17 | #15 OR #16 | 86910 |
| #16 | 'neoadjuvant therap*':ab,ti OR 'neoadjuvant treatment*':ab,ti OR 'neoadjuvant chemoradiotherap*':ab,ti OR 'neoadjuvant chemoradiation treatment*':ab,ti OR 'neoadjuvant chemoradiation*':ab,ti OR 'neoadjuvant chemotherap*':ab,ti OR 'neoadjuvant chemotherapy treatment*':ab,ti OR 'neoadjuvant systemic therap*':ab,ti OR 'neoadjuvant systemic treatment*':ab,ti | 67072 |
| #15 | 'neoadjuvant therapy'/exp | 52270 |
| #14 | #12 OR #13 | 150654 |
| #13 | 'uterine cervical neoplas*':ab,ti OR 'cervical neoplas*':ab,ti OR 'uterine cervical neoplasms':ab,ti OR 'cervix neoplas*':ab,ti OR 'uterine cervical cancer*':ab,ti OR 'cancer of cervix':ab,ti OR 'cervix cancer':ab,ti OR 'uterine cervix tumor':ab,ti OR 'cervical tumo*':ab,ti OR 'cervix neoplasm*':ab,ti OR 'cervix tumorigenesis':ab,ti OR 'cervix uteri tumor':ab,ti OR 'neoplastic cervical':ab,ti OR 'neoplastic cervix':ab,ti OR 'uterine cervical neoplasm*':ab,ti OR 'uterine cervical tumor':ab,ti OR 'uterine cervix neoplas*':ab,ti OR 'uterine cervix tumo*':ab,ti | 11831 |
| #12 | 'uterine cervix tumor'/exp | 148913 |
| #11 | #7 OR #10 | 389660 |
| #10 | #8 OR #9 | 104318 |
| #9 | chemoradiotherap*:ab,ti OR radiochemotherap*:ab,ti OR 'concurrent chemoradiotherap*':ab,ti OR 'synchronous chemoradiotherap*':ab,ti OR 'concurrent radiochemotherap*':ab,ti OR 'concomitant chemoradiotherap*':ab,ti OR 'Chemo-Radiotherapy':ab,ti OR chemoradiation:ab,ti | 77223 |
| #8 | chemoradiotherapy'/exp | 76594 |
| #7 | #3 AND #6 | 377469 |
| #6 | #4 OR #5 | 3884124 |
| #5 | 'drug therap*':ab,ti OR chemotherap*:ab,ti OR pharmacotherap*:ab,ti OR 'pharmacologic therapy':ab,ti | 920759 |
| #4 | 'drug therapy'/exp | 3553442 |
| #3 | #1 OR #2 | 810356 |
| #2 | radiotherap*:ab,ti OR 'radiation therap*':ab,ti OR 'radiation treatment*':ab,ti OR 'targeted radiotherap*':ab,ti OR 'targeted radiation therap*':ab,ti OR 'radiation therapy':ab,ti OR 'x-ray therapy':ab,ti OR 'radioisotope therapy':ab,ti | 445434 |
| #1 | 'radiotherapy'/exp | 696160 |

**PubMed search strategy**

| Search number | Query | Results |
| --- | --- | --- |
| 18 | ((((("Radiotherapy"[Mesh]) OR ((((((((Radiotherap*[Title/Abstract]) OR (Radiation Therap*[Title/Abstract])) OR (Radiation Treatment*[Title/Abstract])) OR (Radiation Treatment*[Title/Abstract])) OR (Targeted Radiation Therap*[Title/Abstract])) OR (radiation therapy[Title/Abstract])) OR (X-ray therapy[Title/Abstract])) OR (radioisotope therapy[Title/Abstract]))) AND (("Drug Therapy"[Mesh]) OR ((((Drug Therap*[Title/Abstract]) OR (Chemotherap*[Title/Abstract])) OR (Pharmacotherap*[Title/Abstract])) OR (pharmacologic therapy[Title/Abstract])))) OR (("Chemoradiotherapy"[Mesh]) OR (((((((((Chemoradiotherap*[Title/Abstract]) OR (Radiochemotherap*[Title/Abstract])) OR (Concurrent Chemoradiotherap*[Title/Abstract])) OR (Synchronous Chemoradiotherap*[Title/Abstract])) OR (Concurrent Radiochemotherap*[Title/Abstract])) OR (Concomitant Chemoradiotherap*[Title/Abstract])) OR (Concomitant Radiochemotherap*[Title/Abstract])) OR (Chemoradiation[Title/Abstract])) OR (Chemo-Radiotherapy[Title/Abstract])))) AND (("Uterine Cervical Neoplasms"[Mesh]) OR ((((((((((((((((((Uterine Cervical Neoplas*[Title/Abstract]) OR (Cervical Neoplas*[Title/Abstract])) OR (Cervix Neoplas*[Title/Abstract])) OR (Cervical Cancer*[Title/Abstract])) OR (Uterine Cervical Cancer*[Title/Abstract])) OR (Cancer of Cervix[Title/Abstract])) OR (Cervix Cancer[Title/Abstract])) OR (uterine cervix tumor[Title/Abstract])) OR (cervical tumo*[Title/Abstract])) OR (cervix neoplasm*[Title/Abstract])) OR (cervix tumorigenesis[Title/Abstract])) OR (cervix uteri tumor[Title/Abstract])) OR (neoplastic cervical[Title/Abstract])) OR (neoplastic cervix[Title/Abstract])) OR (uterine cervical neoplasm*[Title/Abstract])) OR (uterine cervical tumor[Title/Abstract])) OR (uterine cervix neoplas*[Title/Abstract])) OR (uterine cervix tumo*[Title/Abstract])))) AND (("Neoadjuvant Therapy"[Mesh]) OR ((((((((((Neoadjuvant Therap*[Title/Abstract]) OR (Neoadjuvant Treatment*[Title/Abstract])) OR (Neoadjuvant Chemoradiotherap*[Title/Abstract])) OR (Neoadjuvant Chemoradiation Therap*[Title/Abstract])) OR (Neoadjuvant Chemoradiation Treatment*[Title/Abstract])) OR (Neoadjuvant Chemoradiation*[Title/Abstract])) OR (Neoadjuvant Chemotherap*[Title/Abstract])) OR (Neoadjuvant Chemotherapy Treatment*[Title/Abstract])) OR (Neoadjuvant Systemic Therap*[Title/Abstract])) OR (Neoadjuvant Systemic Treatment*[Title/Abstract]))) | 613 |
| 17 | ("Neoadjuvant Therapy"[Mesh]) OR ((((((((((Neoadjuvant Therap*[Title/Abstract]) OR (Neoadjuvant Treatment*[Title/Abstract])) OR (Neoadjuvant Chemoradiotherap*[Title/Abstract])) OR (Neoadjuvant Chemoradiation Therap*[Title/Abstract])) OR (Neoadjuvant Chemoradiation Treatment*[Title/Abstract])) OR (Neoadjuvant Chemoradiation*[Title/Abstract])) OR (Neoadjuvant Chemotherap*[Title/Abstract])) OR (Neoadjuvant Chemotherapy Treatment*[Title/Abstract])) OR (Neoadjuvant Systemic Therap*[Title/Abstract])) OR (Neoadjuvant Systemic Treatment*[Title/Abstract])) | 47,999 |
| 16 | (((((((((Neoadjuvant Therap*[Title/Abstract]) OR (Neoadjuvant Treatment*[Title/Abstract])) OR (Neoadjuvant Chemoradiotherap*[Title/Abstract])) OR (Neoadjuvant Chemoradiation Therap*[Title/Abstract])) OR (Neoadjuvant Chemoradiation Treatment*[Title/Abstract])) OR (Neoadjuvant Chemoradiation*[Title/Abstract])) OR (Neoadjuvant Chemotherap*[Title/Abstract])) OR (Neoadjuvant Chemotherapy Treatment*[Title/Abstract])) OR (Neoadjuvant Systemic Therap*[Title/Abstract])) OR (Neoadjuvant Systemic Treatment*[Title/Abstract]) | 37,075 |
| 15 | "Neoadjuvant Therapy"[Mesh] | 28,847 |
| 14 | ("Uterine Cervical Neoplasms"[Mesh]) OR ((((((((((((((((((Uterine Cervical Neoplas*[Title/Abstract]) OR (Cervical Neoplas*[Title/Abstract])) OR (Cervix Neoplas*[Title/Abstract])) OR (Cervical Cancer*[Title/Abstract])) OR (Uterine Cervical Cancer*[Title/Abstract])) OR (Cancer of Cervix[Title/Abstract])) OR (Cervix Cancer[Title/Abstract])) OR (uterine cervix tumor[Title/Abstract])) OR (cervical tumo*[Title/Abstract])) OR (cervix neoplasm*[Title/Abstract])) OR (cervix tumorigenesis[Title/Abstract])) OR (cervix uteri tumor[Title/Abstract])) OR (neoplastic cervical[Title/Abstract])) OR (neoplastic cervix[Title/Abstract])) OR (uterine cervical neoplasm*[Title/Abstract])) OR (uterine cervical tumor[Title/Abstract])) OR (uterine cervix neoplas*[Title/Abstract])) OR (uterine cervix tumo*[Title/Abstract])) | 107,586 |
| 13 | (((((((((((((((((Uterine Cervical Neoplas*[Title/Abstract]) OR (Cervical Neoplas*[Title/Abstract])) OR (Cervix Neoplas*[Title/Abstract])) OR (Cervical Cancer*[Title/Abstract])) OR (Uterine Cervical Cancer*[Title/Abstract])) OR (Cancer of Cervix[Title/Abstract])) OR (Cervix Cancer[Title/Abstract])) OR (uterine cervix tumor[Title/Abstract])) OR (cervical tumo*[Title/Abstract])) OR (cervix neoplasm*[Title/Abstract])) OR (cervix tumorigenesis[Title/Abstract])) OR (cervix uteri tumor[Title/Abstract])) OR (neoplastic cervical[Title/Abstract])) OR (neoplastic cervix[Title/Abstract])) OR (uterine cervical neoplasm*[Title/Abstract])) OR (uterine cervical tumor[Title/Abstract])) OR (uterine cervix neoplas*[Title/Abstract])) OR (uterine cervix tumo*[Title/Abstract]) | 70,065 |
| 12 | "Uterine Cervical Neoplasms"[Mesh] | 85,769 |
| 11 | ((("Radiotherapy"[Mesh]) OR ((((((((Radiotherap*[Title/Abstract]) OR (Radiation Therap*[Title/Abstract])) OR (Radiation Treatment*[Title/Abstract])) OR (Radiation Treatment*[Title/Abstract])) OR (Targeted Radiation Therap*[Title/Abstract])) OR (radiation therapy[Title/Abstract])) OR (X-ray therapy[Title/Abstract])) OR (radioisotope therapy[Title/Abstract]))) AND (("Drug Therapy"[Mesh]) OR ((((Drug Therap*[Title/Abstract]) OR (Chemotherap*[Title/Abstract])) OR (Pharmacotherap*[Title/Abstract])) OR (pharmacologic therapy[Title/Abstract])))) OR (("Chemoradiotherapy"[Mesh]) OR (((((((((Chemoradiotherap*[Title/Abstract]) OR (Radiochemotherap*[Title/Abstract])) OR (Concurrent Chemoradiotherap*[Title/Abstract])) OR (Synchronous Chemoradiotherap*[Title/Abstract])) OR (Concurrent Radiochemotherap*[Title/Abstract])) OR (Concomitant Chemoradiotherap*[Title/Abstract])) OR (Concomitant Radiochemotherap*[Title/Abstract])) OR (Chemoradiation[Title/Abstract])) OR (Chemo-Radiotherapy[Title/Abstract]))) | 148,135 |
| 10 | ("Chemoradiotherapy"[Mesh]) OR (((((((((Chemoradiotherap*[Title/Abstract]) OR (Radiochemotherap*[Title/Abstract])) OR (Concurrent Chemoradiotherap*[Title/Abstract])) OR (Synchronous Chemoradiotherap*[Title/Abstract])) OR (Concurrent Radiochemotherap*[Title/Abstract])) OR (Concomitant Chemoradiotherap*[Title/Abstract])) OR (Concomitant Radiochemotherap*[Title/Abstract])) OR (Chemoradiation[Title/Abstract])) OR (Chemo-Radiotherapy[Title/Abstract])) | 52,448 |
| 9 | ((((((((Chemoradiotherap*[Title/Abstract]) OR (Radiochemotherap*[Title/Abstract])) OR (Concurrent Chemoradiotherap*[Title/Abstract])) OR (Synchronous Chemoradiotherap*[Title/Abstract])) OR (Concurrent Radiochemotherap*[Title/Abstract])) OR (Concomitant Chemoradiotherap*[Title/Abstract])) OR (Concomitant Radiochemotherap*[Title/Abstract])) OR (Chemoradiation[Title/Abstract])) OR (Chemo-Radiotherapy[Title/Abstract]) | 46,375 |
| 8 | "Chemoradiotherapy"[Mesh] | 20,105 |
| 7 | (("Radiotherapy"[Mesh]) OR ((((((((Radiotherap*[Title/Abstract]) OR (Radiation Therap*[Title/Abstract])) OR (Radiation Treatment*[Title/Abstract])) OR (Radiation Treatment*[Title/Abstract])) OR (Targeted Radiation Therap*[Title/Abstract])) OR (radiation therapy[Title/Abstract])) OR (X-ray therapy[Title/Abstract])) OR (radioisotope therapy[Title/Abstract]))) AND (("Drug Therapy"[Mesh]) OR ((((Drug Therap*[Title/Abstract]) OR (Chemotherap*[Title/Abstract])) OR (Pharmacotherap*[Title/Abstract])) OR (pharmacologic therapy[Title/Abstract]))) | 127,558 |
| 6 | ("Drug Therapy"[Mesh]) OR ((((Drug Therap*[Title/Abstract]) OR (Chemotherap*[Title/Abstract])) OR (Pharmacotherap*[Title/Abstract])) OR (pharmacologic therapy[Title/Abstract])) | 1,930,988 |
| 5 | (((Drug Therap*[Title/Abstract]) OR (Chemotherap*[Title/Abstract])) OR (Pharmacotherap*[Title/Abstract])) OR (pharmacologic therapy[Title/Abstract]) | 609,381 |
| 4 | "Drug Therapy"[Mesh] | 1,511,387 |
| 3 | ("Radiotherapy"[Mesh]) OR ((((((((Radiotherap*[Title/Abstract]) OR (Radiation Therap*[Title/Abstract])) OR (Radiation Treatment*[Title/Abstract])) OR (Radiation Treatment*[Title/Abstract])) OR (Targeted Radiation Therap*[Title/Abstract])) OR (radiation therapy[Title/Abstract])) OR (X-ray therapy[Title/Abstract])) OR (radioisotope therapy[Title/Abstract])) | 393,303 |
| 2 | (((((((Radiotherap*[Title/Abstract]) OR (Radiation Therap*[Title/Abstract])) OR (Radiation Treatment*[Title/Abstract])) OR (Radiation Treatment*[Title/Abstract])) OR (Targeted Radiation Therap*[Title/Abstract])) OR (radiation therapy[Title/Abstract])) OR (X-ray therapy[Title/Abstract])) OR (radioisotope therapy[Title/Abstract]) | 298,208 |
| 1 | "Radiotherapy"[Mesh] | 208,375 |

**Web of science search strategy**

| No. | Query | Results |
| --- | --- | --- |
| 1 | Radiotherapy (Topic) or Radiotherap* (Topic) or Radiation Therap* (Topic) or Radiation Treatment* (Topic) or Targeted Radiotherap* (Topic) or Targeted Radiation Therap* (Topic) or radiation therapy (Topic) or X-ray therapy (Topic) or radioisotope therapy (Topic) | 476688 |
| 2 | Drug Therapy (Topic) or Drug Therap* (Topic) or Chemotherap* (Topic) or Pharmacotherap* (Topic) or pharmacologic therapy (Topic) | 1269005 |
| 3 | #2 AND #1 | 129755 |
| 4 | Chemoradiotherapy (Topic) or Chemoradiotherap* (Topic) or Radiochemotherap* (Topic) or Concurrent Chemoradiotherap* (Topic) or Synchronous Chemoradiotherap* (Topic) or Concurrent Radiochemotherap* (Topic) or Concomitant Chemoradiotherap* (Topic) or Concomitant Radiochemotherap* (Topic) or Chemo-Radiotherapy (Topic) or Chemoradiation (Topic) | 59650 |
| 5 | #3 OR #4 | 167739 |
| 6 | Uterine Cervical Neoplasms (Topic) or Uterine Cervical Neoplas* (Topic) or Cervical Neoplas* (Topic) or Cervix Neoplas* (Topic) or Cervical Cancer* (Topic) or Uterine Cervical Cancer* (Topic) or Cancer of Cervix (Topic) or Cervix Cancer (Topic) or uterine cervix tumor (Topic) or cervical tumo* (Topic) or cervix neoplasm* (Topic) or cervix tumorigenesis (Topic) or cervix uteri tumor (Topic) or neoplastic cervical (Topic) or neoplastic cervix (Topic) or uterine cervical neoplasm* (Topic) or uterine cervical tumor (Topic) or uterine cervix neoplas* (Topic) or uterine cervix tumo* (Topic) | 128231 |
| 7 | Neoadjuvant Therapy (Topic) or Neoadjuvant Therap* (Topic) or Neoadjuvant Treatment* (Topic) or Neoadjuvant Chemoradiotherap* (Topic) or Neoadjuvant Chemoradiation Therap* (Topic) or Neoadjuvant Chemoradiation Treatment* (Topic) or Neoadjuvant Chemoradiation* (Topic) or Neoadjuvant Chemotherap* (Topic) or Neoadjuvant Chemotherapy Treatment* (Topic) or Neoadjuvant Systemic Therap* (Topic) or Neoadjuvant Systemic Therap* (Topic) | 65992 |
| 8 | #5 AND #6 AND #7 | 1240 |
